# Microencapsulated Pomegranate Reverts High-Density Lipoprotein (HDL)-Induced Endothelial Dysfunction and Reduces Postprandial Triglyceridemia in Women with Acute Coronary Syndrome

**DOI:** 10.3390/nu11081710

**Published:** 2019-07-25

**Authors:** Diego Estrada-Luna, Elizabeth Carreón-Torres, Rocío Bautista-Pérez, Gabriel Betanzos-Cabrera, Alan Dorantes-Morales, María Luna-Luna, Jesús Vargas-Barrón, Ana María Mejía, José Manuel Fragoso, Karla Carvajal-Aguilera, José J. García-Trejo, Gilberto Vargas-Alarcón, Óscar Pérez-Méndez

**Affiliations:** 1 Department of Molecular Biology, Instituto Nacional de Cardiología “Ignacio Chávez”, 14080 Mexico City, Mexico; 2 School of Engineering and Sciences Campus Queretaro, Tecnologico de Monterrey, 76130 Queretaro, Mexico; 3 Blood Bank, Instituto Nacional de Cardiología “Ignacio Chávez”, 14080 Mexico City, Mexico; 4 Laboratory of Experimental Nutrition, Instituto Nacional de Pediatría, 04530 Mexico City, Mexico; 5 Department of Biology, School of Chemistry, Universidad Nacional Autónoma de México (U.N.A.M.), 04510 Mexico City, Mexico

**Keywords:** microencapsulated pomegranate, high-density lipoproteins, endothelial dysfunction, acute coronary syndrome in women, postprandial state, atherosclerosis

## Abstract

(1) Background: the composition of high-density lipoproteins (HDL) becomes altered during the postprandial state, probably affecting their functionality vis-à-vis the endothelium. Since acute coronary syndrome (ACS) in women is frequently associated with endothelial dysfunction, it is likely that HDL are unable to improve artery vasodilation in these patients. Therefore, we characterized HDL from women with ACS in fasting and postprandial conditions. We also determined whether microencapsulated pomegranate (MiPo) reverts the HDL abnormalities, since previous studies have suggested that this fruit improves HDL functionality. (2) Methods: Eleven women with a history of ACS were supplemented daily with 20 g of MiPo, for 30 days. Plasma samples were obtained during fasting and at different times, after a lipid load test to determine the lipid profile and paraoxonase–1 (PON1) activity. HDL were isolated by sequential ultracentrifugation to determine their size distribution and to assess their effect on endothelial function, by using an in vitro model of rat aorta rings. (3) Results: MiPo improved the lipid profile and increased PON1 activity, as previously reported, with fresh pomegranate juice. After supplementation with MiPo, the incremental area under the curve of triglycerides decreased to half of the initial values. The HDL distribution shifted from large HDL to intermediate and small-size particles during the postprandial period in the basal conditions, whereas such a shift was no longer observed after MiPo supplementation. Consistently, HDL isolated from postprandial plasma samples hindered the vasodilation of aorta rings, and this endothelial dysfunction was reverted after MiPo consumption. (4) Conclusions: MiPo exhibited the same beneficial effects on the lipid profile and PON1 activity as the previously reported fresh pomegranate. In addition, MiPo supplementation reverted the negative effects of HDL on endothelial function generated during the postprandial period in women with ACS.

## 1. Introduction

High-density lipoproteins (HDL) are heterogeneous macromolecular complexes that are inversely correlated with the risk of cardiovascular disease; HDL promote reverse transport of cholesterol (RTC) and possess antithrombotic, anti-inflammatory and antioxidant properties [1]. However, therapies focused on increasing HDL-cholesterol (HDL-C) plasma levels have not been associated with a reduction in cardiovascular risk. Therefore, HDL quality and functionality seems to be more important than HDL quantity (estimated by HDL-C) in reducing the incidence of cardiovascular events [2].

HDL may be classified into five subclasses, HDL2b, HDL2a, HDL3a, HDL3b and HDL3c, based on their size [3]. These HDL subclasses differ in terms of lipid and protein composition, and such differences determine the capacity of these lipoproteins to carry metabolites, small RNAs, and, particularly, bioactive molecules that may be related to their antiatherogenic properties [4]. Some authors have suggested that HDL quality is highly dependent on their structure [3,4], and the modification of their chemical composition is inversely related to the generation of dysfunctional HDL particles [4,5]. In this context, previous studies have shown that postprandial hypertriglyceridemia affects the HDL structure [6]. Also, different properties of HDL may be affected during the postprandial state, such as cholesterol efflux [7], paraoxonase-1 (PON1) activity [8], and the capacity to induce the expression and activation of endothelial nitric oxide synthase (eNOS) [9].

Some authors have reported a higher prevalence of plaque erosion and coronary vasospasm in women than men. These signs correlate with endothelial dysfunction [10,11] due to a decrease in nitric oxide (NO) availability, which contributes to coronary heart disease (CHD) progression. Since HDL participate in eNOS regulation [12,13], it is likely that these lipoproteins are dysfunctional in terms of their favorable effect on endothelial cells, particularly during the postprandial state in women with CHD.

Currently, there are therapies focused on improving HDL quality and functionality that include the use of functional foods. In vitro and in vivo studies have demonstrated that pomegranate juice reduced the plasma total cholesterol, triglycerides and low-density lipoprotein-cholesterol (LDL-C) levels, and particularly induced an increase in HDL-C plasma levels and serum PON1 activity [14,15,16,17]. These properties have been attributed to pomegranate compounds such as polyphenols, anthocyanins, ellagitannins and punicic acid [18], which should be absorbed and transported, probably by HDL, in plasma during the postprandial state. In this context, pomegranate may improve the quality of HDL vis-à-vis the endothelial function during postprandial conditions. Therefore, the aim of this study was to analyze the effect of a daily supplementation of microencapsulated pomegranate (MiPo) on HDL generated during the postprandial period, in women with a history of acute coronary syndrome (ACS).

## 2. Materials and Methods 

### 2.1. Patients 

This study included 11 women diagnosed with ACS from the Instituto Nacional de Cardiología “Ignacio Chavez”. Inclusion criteria were as follows: presence of ACS in the last year, absence of dysthyroidism, liver disease, autoimmune or congenital heart disease. Exclusion criteria included tobacco smoking, alcohol consumption, surgery in the last 6 weeks, or intolerance to MiPo. Patients completed a survey regarding medical history, lifestyle factors, medication, and diet habits, which included a 24 h questionnaire. Anthropometric determinations were performed under standardized procedures. For some determinations, we included a group of women (*n* = 6), normotensive and normoglycemic, who had neither a personal nor family history of coronary heart disease.

The study was performed in accordance with the appropriate version of the Declaration of Helsinki and approved by the Ethics Committee from the Instituto Nacional de Cardiología “Ignacio Chávez” with the registration number 16–969. All the patients gave their written informed consent prior to the study. 

### 2.2. Preparation of MiPo

Pomegranates from Valle de Mezquital, Hidalgo, Mexico were handpicked, washed and peeled. Fresh pomegranate arils were ground in a laboratory mill and sieved to remove particles larger than 0.5 mm. The resulting liquid was dried at 50 ± 0.5 °C. The dried material was ground again to obtain a fine powder. Subsequently, an extraction was performed with ethanol:water at a 1:1 (*v*/*w*) ratio and the extract was allowed to stand for 2 h at room temperature. The extract was then filtered through a glass microfiber filter paper. The filtered extract was evaporated using a rotary evaporator at 50 °C to remove ethanol.

Maltodextrin–dextrose equivalent 16.5–19.5 (Amfher Foods, S.A. de C.V., Mexico City, Mexico) and gum Arabic (Sigma–Aldrich Chemical Co., St. Louis, MO, USA) were selected as coating materials; both were dispersed individually in water until reaching 10.0% solid content. The solution of coating materials was prepared by mixing maltodextrin and gum Arabic at a 4:1 (*v*/*v*) ratio. The coating solution was combined with the aril extract and homogenized for 10 min at 8000 rpm, 60 °C in a magnetic stirrer. Then, the homogenate was spray-dried in a Büchi B–191 Mini Spray-Dryer and fed at room temperature with an inlet air temperature of 110 °C and a pump flow of 600 mL/min. The microencapsulated powder showed great stability and was stored in darkness at room temperature until its use. Once the conditions were standardized, microencapsulated pomegranate was produced on an industrial scale by Granding International, S.A. de C.V., Jiutepec Morelos, Mexico. 

### 2.3. Dietary Interventions

The current study included three visits; during the first visit, one week previous to the supplementation with MiPo, patients were instructed to follow an isocaloric diet personally designed to reach a balance intake of 60, 25, and 15% of total calories from carbohydrates, lipids and proteins, respectively. At the second visit, patients underwent a lipid load test consisting in a 1000-kcal lunch containing 75 g of fat, to be eaten within 20 min to induce postprandial hypertriglyceridemia [6]; patients were not allowed to eat or drink for 8 h, except water. To minimize physical activity, all patients stayed in a metabolic room throughout the testing time. Then, patients consumed 20 g of MiPo dissolved in 250 mL of water daily, for 30 days. At the third visit, after the MiPo supplementation, patients repeated the lipid load test. Each patient was provided with a written set of guidelines and record sheets for food intake.

At every visit, the anthropometric characteristics were recorded, and patients completed a 24-h food frequency reminder. In order to be sure that the diet and exercise preceding the trials would not affect biochemical parameters, participants were instructed to refrain from excessive exercise. 

### 2.4. Laboratory Analysis

After 12 h of overnight fasting, an intravenous catheter was inserted into a forearm vein for blood collection. Twenty mL of blood samples were drawn in EDTA or dry tubes, in fasting conditions and then at 4 and 8 h after the meal during the second and third visits. Blood samples were centrifuged for 15 min at 2500 RPM within 15 min after collection. Plasma and serum were separated into 500 µL aliquots, and then either immediately analyzed or frozen at −80 °C until analysis. Glucose, cholesterol, and triglycerides (Tg) plasma concentrations were determined by commercially available enzymatic/colorimetric assays (Randox Labs. Ltd., Crumlin, Co., Antrim, Northern Ireland). The phosphotungstic acid-Mg^+2^ method was used to precipitate apo B-containing lipoproteins before quantifying HDL-cholesterol (HDL-C), HDL-triglycerides (HDL-Tg) and HDL-phospholipids (HDL-Pho) concentrations. Plasma lipid concentrations were determined within 24 h after collection. LDL-C was estimated in samples with Tg < 400 mg/dL using the Friedewald method. The incremental area under the curve (iAUC) of Tg was determined using a conventional trapezoid method to compare postprandial response between pre-and post-treatment (response to fat load test) [6]. 

### 2.5. HDL Subclasses Assessment

HDL were separated by ultracentrifugation using a solution with a density of 1.21 g/mL, as previously described [19]. Recovered HDL were dialyzed against 0.09 M Tris/0.08 M boric acid/3 mM EDTA buffer, pH 8.4. To further separate the recovered fractions by their hydrodynamic diameter, 25 µg of HDL protein was run in a non-denaturing 3–30% gradient polyacrylamide gel electrophoresis [20]. Gels were scanned and stained with coomassie blue R–250 in a GS–800 Calibrated Densitometer (BioRad Laboratories, Hercules, CA, USA) to detect proteins. The sizes of HDL (Stoke’s diameter) subclasses was determined by densitometric analysis, considering the following size intervals: HDL 3c, 7.94–8.45 nm; HDL 3b, 8.45–8.98 nm; HDL 3a, 8.98–9.94 nm; HDL 2a, 9.94–10.58 nm; HDL 2b, 10.58–13 nm. To delimit HDL subclasses, we used globular proteins as a diameter reference (thyroglobulin 17 nm; ferritin 12.2 nm; catalase 10.4 nm; lactate dehydrogenase 8.2 nm; albumin 7.1 nm; high molecular weight calibration kit, Amersham Pharmacia Biotech, Buckinghamshire, UK). Each subclass was measured using VisionWorks version 8.20 and expressed as the percentage of total HDL area of the densitogram [20]. 

### 2.6. Vascular Reactivity of Aorta Rings 

The effect of HDL on endothelial function was evaluated using aorta rings from Wistar rats in an organ bath, as previously described [21]. Aorta rings were incubated with isolated HDL to a final concentration of 50 mg/dL of cholesterol in Krebs solution (NaCl 118.1 mM, KCl 4.7 mM, MgSO_4_ 1.2 mM, KH_2_PO_4_ 1.20 mM, CaCl_2_ 2.5 mM, NaHCO_3_ 25.0 mM, glucose 11.1 mM at pH 7.4), continuously bubbled with 95% O_2_, 5% CO_2_, at 37 °C, 1 h previous to the test. After an equilibrium period of 2 h, aorta rings were pre-contracted with 3 × 10^−4^ M phenylephrine. The endothelium-mediated relaxation (vasodilation) was evaluated by a dose-response curve to acetylcholine (5 × 10^−9^ to 8 × 10^−7^ M). Contractions were measured isometrically with an FT–03 Grass Force Displacement Transducer and recorded on a Grass Polygraph (Model 7D, Grass Medical Instruments, Quincy, MA, USA). All experiments were run in duplicate, and the relaxation of aorta rings was expressed as the percentage of pre-contraction with phenylephrine [21]. 

### 2.7. Paraoxonase–1 (PON1) Activity 

PON1 was determined using phenylacetate as a substrate [22]. Initial rate of hydrolysis were measured spectrophotometrically at 270 nm (UV-VIS Beckman Coulter, Brea, CA, USA). The assay mixture included 1 mM phenylacetate and 0.9 mM CaCl_2_ in 20 mM Tris-HCl, pH 8 and 10 µL serum (diluted 1:40). The ΔA270 nm for the reaction was 1310 M^−1^ cm^−1^. The enzyme activity was expressed as the number of micromoles of phenylacetate hydrolyzed per minute, per milliliter of serum [22].

### 2.8. Data Analysis 

Unless otherwise indicated, data are presented as median and interquartile ranges. Wilcoxon’s paired test was used to compare medians of pre- and post-supplementation results. The normal distribution of the vasorelaxation percentages was verified by the Kolmogorov–Smirnov test; since these data were normally distributed, a Student’s paired *t* test was used to compare the pre- and post-supplementation results obtained with each acetylcholine concentration. Statistical significance was defined as *p* ≤ 0.05. All statistics were performed with SPSS 24.0 software (SPSS Inc. IBM, Chicago, IL, USA). 

## 3. Results

### 3.1. Study Population

The clinical characteristics and the caloric intake balance of the patients with ACS included in the study are shown in Table 1. The caloric intake was controlled and remained similar to the initial conditions after the supplementation with the MiPo. In addition, women with ACS showed a slight but significant decrease of 2.27% in waist circumference (*p* ≤ 0.05) after 30 days of MiPo consumption. The use of statins, beta-blockers and angiotensin converting enzyme (ACEI) inhibitors were maintained during the consumption of MiPo. As a reference for some iAUC and vascular reactivity of aorta rings in the presence of HDL, we included a group of six women without ACS, so-called non-ACS, whose anthropometric and biochemical data are shown in Supplementary Materials Tables S1 and S2.

### 3.2. Biochemical Parameter Profile

Before the pre-supplementation with MiPo, triglyceride concentrations significantly increased by about 80% and 61% at 4 h and 8 h after the lipid load test, respectively, as compared with fasting conditions. Similarly, after the supplementation, there were significant increases in triglycerides, 97% at 4 h and 12% at 8 h. Post-MiPo values of postprandial triglyceridemia were 16%, 8% and 42% significantly lower at 0, 4, and 8 h, respectively, than the corresponding times in the pre-supplementation conditions. Total cholesterol levels also decreased by between 8% and 15% after treatment with MiPo at all of the three times registered (Table 2). The LDL-C fraction was the most importantly reduced with MiPo treatment at any time of the curve; this fraction significantly decreased by 27% in fasting conditions, as well as by 36% at 4 h and 35% at 8 h after the meal, when compared with the pre-supplementation conditions. Glucose levels showed a tendency toward lower fasting values after MiPo consumption, but the difference did not reach statistical significance (Table 2). Similar tendencies were observed in the group of non-ACS women, even if for some parameters the differences before and after MiPo did not reach statistical significance. 

The incremental area under the curve (iAUC) of triglycerides represents the net increase during the postprandial period, independently of the fasting plasma levels. The median of the iAUC of triglycerides significantly decreased by 9% after MiPo consumption (Table 2). Similar to the ACS patients, in the non-ACS group, the median of the iAUC after the supplementation with MiPo was also lower (−14%) than that of the basal conditions. However, the difference did not reach statistical significance (Supplementary Materials Table S2).

### 3.3. HDL Lipid Profile

Fasting HDL-C plasma levels significantly increased by 11% after 30 days of MiPo supplementation (Table 2). Along the postprandial period, there was a significant decrease in HDL-C plasma concentration in both pre-and post-supplementation conditions; however, HDL-C concentrations were higher in the latter than in the former condition, at any point of the curve (Table 2). Concomitantly, HDL-Tg plasma concentrations significantly increased at 8 h of postprandial state after the MiPo consumption, whereas there were no significant postprandial modifications of HDL-Tg before supplementation (Table 2). Finally, HDL-Pho concentrations at 8 h after the meal in the post-supplementation condition were about 6% higher than the fasting values (Table 2) and 15% higher when compared with the corresponding time (8 h) before the supplementation.

We further determined the HDL-Tg/HDL-Pho and HDL-C/HDL-Pho ratios as markers of HDL lipid composition [23] (Table 2). In post-supplementation conditions, we found a significant decrease of 6 and 21% in the HDL-C/HDL-Pho ratio (Table 2) at 4 h and 8 h after the lipid load test, respectively, compared with fasting values. In contrast, the HDL-Tg/HDL-Pho ratio remained unchanged at any time of the postprandial state in both pre-and post-supplementation conditions (Table 2). 

### 3.4. HDL Size Distribution

We look further to determine the structure of HDL during the supplementation with MiPo (Table 3); comparing fasting conditions, HDL2b was slightly but significantly higher after MiPo than before the supplementation. Likewise, the relative proportion of HDL2b increased by 25%, with a concomitant decrease in HDL3a and HDL3b, at 8 h after the meal during the basal conditions (*p* ≤ 0.05 for all comparisons), but such postprandial changes were no longer observed after MiPo treatment (Table 3).

### 3.5. PON1 Activity

**Figure 1:**
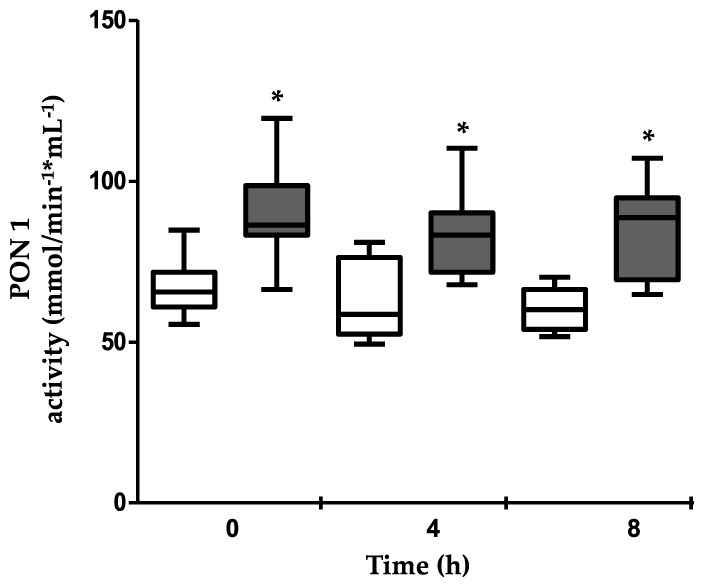
Effect of microencapsulated pomegranate (MiPo) on paraoxonase–1 (PON1) activity in the serum of acute coronary syndrome (ACS) women. PON1 activity was determined at the basal conditions (white boxes) and after 30 d of supplementation with MiPo (gray boxes). The measurements were taken at fasting and at 4 and 8 h after the lipid load test. The median is represented by the horizontal line and boxes represent the interquartile interval. * Wilcoxon’s test *p* ≤ 0.01, between pre- and post-supplementation.

PON1 activity was measured using phenylacetate as a substrate, as described in the Methods section; MiPo supplementation induced a 20% to 29% increase in PON1 activity at fasting, 4 h and 8 h, as compared with the pre-treatment conditions (*p* ≤ 0.01 for all) (Figure 1).

### 3.6. Effect of MiPo on Aortic Rings Incubated with HDL of ACS Women

**Figure 2:**
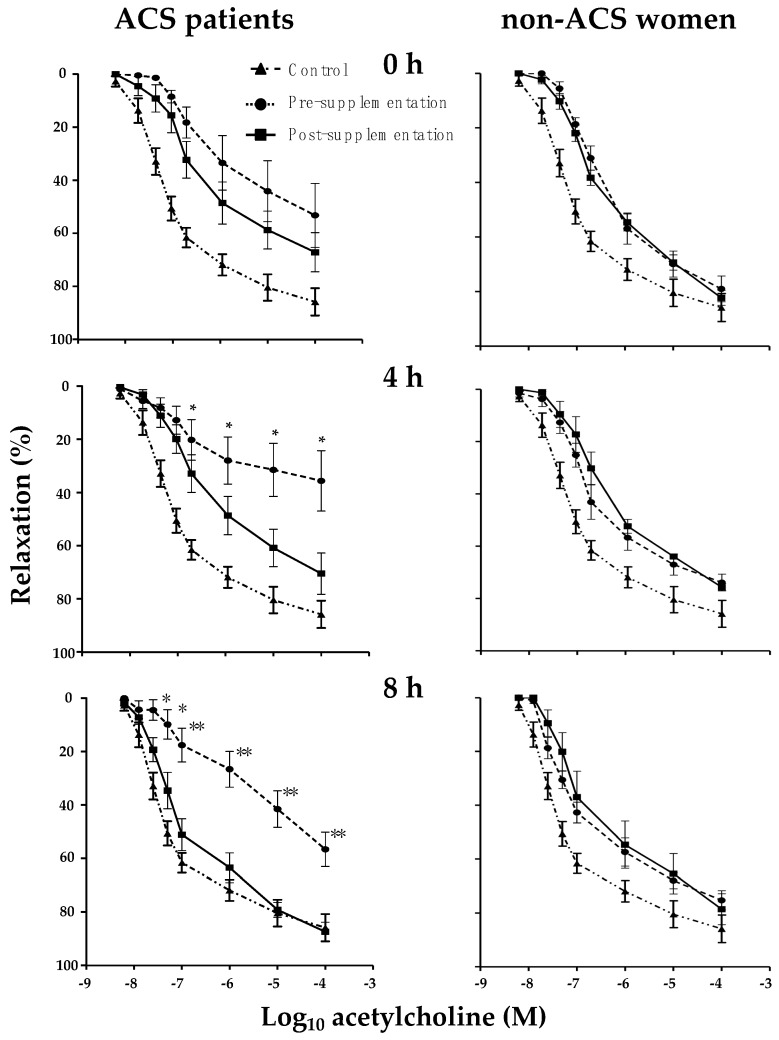
Effects of MiPo on endothelial-dependent relaxation of rat aortic rings incubated with high-density lipoproteins (HDL) from ACS patients (*n* = 11) and the reference group of non-ACS women (*n* = 6). Curves represent the % of relaxation of aorta rings, pre-contracted with phenylephrine and incubated with HDL, from before (black circles) and after 30 days of supplementation with MiPo (black squares) in function of the logarithm of acetylcholine concentration. Fasting conditions, and 4 h and 8 h after the lipid load test, are shown in the top, middle and bottom graphs, respectively. Each point represents the mean and standard error of the mean (SEM). A curve of 11 independent experiments using aorta rings incubated in the absence of HDL has been included in every graph (black triangles). Pre- and post-supplementation paired Student *t* test, * *p* ≤ 0.05 and ** *p* ≤ 0.01.

In order to explore whether the slight but significant structural changes of HDL had an impact on the functionality of these lipoproteins, we determined the endothelial-dependent vasodilation of rat aorta rings in the presence of HDL. As shown in Figure 2, the vasodilation of rings incubated with HDL isolated from plasma after MiPo supplementation was more significant than that of rings incubated with HDL obtained from plasma before the intervention. The vasodilation tended to be lower before MiPo than after the intervention, but the differences did not reach statistical significance at any concentration of acetylcholine (Figure 2). In contrast, the vasodilation at 4 h in the presence of HDL isolated after the supplementation was significantly higher compared to HDL isolated before the MiPo, particularly in the range of concentrations from 10^−7^ to 10^−4^ M of acetylcholine (*p* ≤ 0.05) (Figure 2). At 8 h after the lipid load test, the effects of HDL post-supplementation on vasodilation were more pronounced than those observed at 4 h; with HDL obtained after intervention, the percentage of vasodilation was higher than that before MiPo, at any concentration of acetylcholine (Figure 2). In contrast, HDL generated during the postprandial state in non-ACS women did not limit the vasodilation, either before or after MiPo supplementation (Figure 2)

## 4. Discussion

In the current study, we tested a new formulation of pomegranate in women with acute coronary syndrome (ACS) in order to evaluate HDL functionality generated during the postprandial state, along with its effects on other cardiovascular risk factors. 

MiPo consumption resulted in a reduction of BMI, waist circumference, systolic blood pressure, total cholesterol, triglycerides and LDL-C levels, as well as an increase in PON1 activity and changes of the lipid profile in HDL. Although it is still controversial [24,25], some studies have shown that pomegranate induces an increase in HDL-C and a decrease in triglycerides and LDL-C plasma levels, as observed in our study. As a possible mechanism that leads to these beneficial effects, it has been proposed that some compounds of pomegranate may activate PPAR-α and PPAR-γ [26,27,28,29]. Independently of the involved mechanisms, these results clearly indicate that the MiPo conserved the beneficial properties of the fresh juice [14,15]. The microencapsulated form has several advantages over the fresh juice; pomegranate is a seasonal fruit, and as a consequence it cannot be continuously consumed. In this regard, the microencapsulated form allows a wider use of pomegranate as a functional food throughout the year. In addition, MiPo has a long shelf life, low stock volumes, thus it is easier to distribute than the fresh fruit, even in regions where the fruit is not cultivated. 

It has been proposed that coronary heart disease is a postprandial phenomenon [30]. In agreement with this idea, postprandial triglyceridemia is an independent risk factor of cardiovascular events [31]. In order to explore whether MiPo provides beneficial effects during the postprandial state, we determined the incremental area under the curve (iAUC) of triglycerides. This parameter is a better marker of postprandial modifications of triglycerides metabolism than the total area under the curve [6,32]. Our data clearly demonstrated a very important decrease in the iAUC in patients with ACS after supplementation with MiPo. Considering that pomegranate compounds activate PPAR-α [26] and PPAR-γ [27], the improvement in iAUC observed after MiPo may be related to an overexpression of LPL activity. However, a decreased intestinal absorption of triglycerides cannot be discarded. Specific studies are needed to elucidate the mechanisms implicated in the improvement in postprandial triglyceridemia.

We particularly focused on HDL during the postprandial state, as described above; despite the limited number of included patients, endothelial dysfunction was highly evident when aorta rings were incubated with HDL generated during the postprandial period. Previous studies suggest that HDL functionality is dependent on the size and composition of the particles [5,6,23,33]. In this context, previous studies demonstrated that HDL became remodeled during the postprandial state, and such remodeling was different in subjects with a higher risk of cardiovascular disease than in control subjects [6]. Therefore, we explored whether MiPo could induce changes in postprandial HDL remodeling and whether such changes could be associated with more functional particles. Our results clearly showed that in basal conditions, there were some slight but significant modifications of the relative proportion of HDL subclasses after a high-fat meal. Such modifications were no longer observed after MiPo supplementation. Accordingly, when aorta rings were co-incubated with HDL isolated before the intervention, we demonstrated an endothelial dysfunction. Moreover, the deleterious effects of HDL obtained from plasma of women with ACS were reverted by MiPo supplementation. Such postprandial vascular dysfunction was not observed with HDL isolated from plasma of non-ACS women; in this group, the response to increasing concentrations of acetylcholine was very close to the control aorta rings without HDL. These results indicate that HDL from non-ACS women showed a lack of the deleterious properties of the lipoproteins isolated from ACS patients. As expected, the supplementation with MiPo did not have any supplementary beneficial effect on vascular dependent vasorelaxation during the postprandial period. Therefore, HDL-mediated endothelial dysfunction is a particular characteristic of women who have undergone a previous ACS event. To our knowledge, this the first report showing a postprandial dysfunctionality of HDL vis-à vis the endothelium in patients with a history of ACS. 

The HDL-mediated endothelial dysfunction before the supplementation strongly suggests that the structure of these lipoproteins became modified during the postprandial state; HDL size distribution indeed shifted from large HDL2b to medium/small particles (HDL3a and HDL3b) after 8 h since a high-fat meal. Conversely, the lipid composition of the overall HDL subclasses, as determined by the HDL-Tg/Pho and HDL-C/Pho ratios, remained unchanged. Previous studies have demonstrated that the relative proportion of small HDL3c decreased in normal individuals, with concomitant triglyceride and cholesterol enrichments after a high-fat meal [6]. Therefore, it is likely that the re-structuration of HDL during the postprandial period in ACS women is unpaired, and contributes to their deleterious effects on endothelial function. This possibility is congruent with the structure of HDL in the postprandial state after the treatment with MiPo; no changes in HDL size distribution were observed, but HDL3c tended to increase, as previously observed in control subjects [6]. Therefore, MiPo seems to improve HDL postprandial modifications in ACS women, thus inhibiting the deleterious effects of these lipoproteins on endothelial function.

PON1 is an enzyme carried in plasma by HDL that confers to these lipoproteins most of their antioxidant properties [17,34]. PON1 was improved after MiPo, but the activity remains constant during the postprandial period, either before or after the supplementation. These results indicate that HDL remodeling did not modify the affinity of the lipoproteins for the enzyme during postprandial periods. The increase in the PON1 activity after MiPo was probably mediated by a higher synthesis of the protein, as previously suggested [15,27].

We observed a significant increase in HDL-Pho at 8 h after the lipid load test at the end of the post-MiPo supplementation. Considering that HDLs are synthesized by both the liver and gut [3,35], we propose that MiPo enhances the intestinal HDL synthesis, probably via the activation of PPARs [26,27,28,29] or activation of the ATP-binding cassette class A member 1 (ABCA–1) [36]. In addition, it has been demonstrated that some dietary antioxidant and hydrophobic compounds, such as zeaxanthin and lutein, require HDL for intestinal absorption [37]. Therefore, some potentially active hydrophobic compounds of pomegranate, such as punicic acid, could be absorbed via intestinal HDL. Since HDL may be internalized in endothelial cells to deliver their components within the cell [13], the active compounds of pomegranate carried by intestinal HDL may be vectored to the intracellular compartments via these lipoproteins. The particular compounds of pomegranate that may be vectored and internalized in peripheral cells by intestinal HDL is a topic that deserves to be explored in future studies.

As limitations of this study, we recognize that we only included women as well as a low number of individuals. In addition, we did not analyze the balance of saturated/unsaturated fatty acids [38] and other lipids such as sphingomyelin [33,39], which may determine the functionality of HDL. Therefore, future studies should explore whether the beneficial effects of pomegranate are extrapolative to men, and whether other lipids within HDL are modified by the consumption of this fruit. 

## 5. Conclusions

The consumption of microencapsulated pomegranate is able to revert the deleterious effects of the HDL generated during postprandial periods in women with ACS, improving the endothelial function, the iAUC of triglycerides and some other biochemical parameters associated with cardiovascular risk.

## Figures and Tables

**Table 1:** Anthropometric characteristics of the included acute coronary syndrome (ACS) patients and nutrients intake before and after the supplementation with microencapsulated pomegranate (MiPo).

Parameters	Pre-Supplementation *n* = 11	Post-Supplementation *n* = 11	*p* Value *
Age (years)	51.6 (41.2–60.98)		
BMI (kg/m^2^)	26.96 (24.61–29.32)	26.37 (24.06–28.68)	0.105
Waist circumference (cm)	92.45 (85.41–99.49)	91.45 (84.36–98.58)	0.009
Systolic BP (mmHg)	128.3 (113.5–143.1)	121.5 (112.8–130.2)	0.130
Diastolic BP (mmHg)	72.1 (62.1–82.1)	72.4 (65.7–79.1)	0.404
Statins (*n*)	8		
Beta blockers (*n*)	6		
ACEI (*n*)	3		
Nutrient intake:			
Carbohydrates (%)	51.4 (49.7–53.1)	52.29 (50.65–53.94)	0.640
Lipids (%)	20.27 (18.61–21.93)	20.58 (19.1–22.07)	0.705
Protein (%)	28.65 (27.88–29.42)	28.47 (26.76–30.19)	0.810
Calories (kcal)	1869.34 (1761.16–1977.52)	1880.22 (1749.86–2010.58)	0.705

Data are expressed as median (interquartile range). * Wilcoxon test. ACEI: angiotensin converting enzyme inhibitors. BMI: body mass index. BP: Blood pressure.

**Table 2:** Effects of MiPo supplementation on plasma lipids and glucose concentrations.

Parameter		Sample Time (*n* = 11)		
		0 h	4 h	8 h
Glucose (mg/dL)	Pre	88.3 (76.6–125.9)	83.2 (67.9–113.9)	88.9 (76.3–110.4)
	Post	79.7 (73.2–89.0)	88.0 (78.7–95.3)	89.2 (78.2–92.1)
Total cholesterol (mg/dL)	Pre	157.6 (146.5–219.8)	150.9 (139.9–245.8)	141.4 (130.2–254.4)
	Post	133.2 (87.1–140.7) ^a^	138.9 (127.2–149.0) ^b^	128.4 (103.3–148.6) ^c^
LDL-C (mg/dL)	Pre	85.8 (69.1–161.1)	73.4 (56.1–160.4)	74.2 (52.1–180.0)
	Post	62.7 (34.1–88.5) ^a^	46.9 (40.6–63.6) ^b^	48.2 (34.2–65.3) ^c^
Triglycerides (mg/dL)	Pre	125.4 (119.4–182.3)	226.6 (190.0–344.2) ^a^	202.2 (156.6–322.9) ^a,b^
	Post	105.1 (83.5–120.7) ^a^	207.5 (112.5–247.4) ^b,d^	117.2 (92.3–197.9) ^c,d,e^
iAUC (h × mg/dL)	Pre	419.0 (315.3–1011.7)		
	Post	382.8 (107.3–563.0) ^f^		
HDL-C (mg/dL)	Pre	39.4 (35.7–56.7)	38.0 (31.4–47) ^a^	36.6 (33.2–49.2) ^a^
	Post	43.9 (40.2–62.6) ^a^	39.4 (35.8–53.3) ^d^	39.3 (36.8–49.3) ^c,d^
HDL-Tg (mg/dL)	Pre	17.0 (14.6–25.5)	20.1 (15.8–28.0)	25.3 (16.3–30.8)
	Post	19.8 (19.4–31.4)	22.9 (20.3–30.8)	29.8 (23.9–36.7) ^c,d,e^
HDL-Pho (mg/dL)	Pre	89.4 (75.4–115.4)	93.2 (75.4–103.1)	88.6 (72.1–122.0)
	Post	95.9 (80.8–113.0)	90.2 (80.0–103.3)	101.6 (91.2–129.3) ^c,d,e^
Ratios:				
HDL-C/HDL-Pho	Pre	0.489 (0.399–0.559)	0.433 (0.388–0.464)	0.424 (0.388–0.464)
	Post	0.460 (0.421–0.595)	0.429 (0.395–0.499) ^d^	0.363 (0.337–0.447) ^d,e^
HDL-Tg/HDL-Pho	Pre	0.217 (0.146–0.287)	0.232 (0.166–0.301)	0.259 (0.201–0.339)
	Post	0.255 (0.194–0.288)	0.234 (0.201–0.396)	0.278 (0.208–0.375)

Data are expressed as median (interquartile range). Wilcoxon test ^a^ *p* < 0.05 vs. 0 h pre-supplementation. ^b^ *p* < 0.05 vs. 4 h pre-supplementation. ^c^ *p* < 0.05 vs. 8 h pre-supplementation. ^d^ *p* < 0.05 vs. 0 h post-supplementation. ^e^ *p* < 0.05 vs. 4 h post-supplementation. iAUC: Incremental area under the curve. LDL-C: Low-density lipoprotein-cholesterol. HDL-C: High-density lipoprotein-cholesterol. HDL-Tg: HDL-triglycerides. HDL-Pho: HDL-phospholipids.

**Table 3:** Effects of MiPo supplementation on high-density lipoproteins (HDL) size distribution, determined by protein.

HDL Subclasses (%)		Sample Time (*n* = 11)		
		0 h	4 h	8 h
			*n* = 11	
HDL2b	Pre	30.8 (15.9–35.0)	31.5 (16.9–40.3) ^a^	38.6 (18.8–42.3) ^a^
	Post	31.5 (17.3–40.3) ^a^	32.8 (19.3–40.7) ^b^	31.7 (18.2–42.1)
HDL2a	Pre	14.3 (10.9–16.4)	15.0 (10.9–16.6)	16.0 (12.5–18.7)
	Post	15.0 (12.3–17.9)	14.6 (12.4–16.6)	14.7 (10.6–16.9)
HDL3a	Pre	31.0 (27.8–35.9)	29.2 (27.3–33.5)	28.3 (25.9–32.8) ^a^
	Post	27.7 (26.5–34.1) ^a^	29.5 (24.7–34.1)	29.2 (26.0–33.3)
HDL3b	Pre	16.9 (12.5–21.8)	14.1 (10.2–21.5)	10.4 (7.8–21.2) ^a^
	Post	14.7 (10.0–21.8)	15.7 (8.2–25.6)	15.3 (8.8–23.2)
HDL3c	Pre	6.2 (4.6–12.3)	6.4 (3.4–19.3)	4.4 (1.3–15.1)
	Post	5.1 (2.6–10.8)	5.5 (2.3–16.8)	7.9 (1.2–21.3) ^c^

Data are expressed as the median (interquartile range) percentage of protein associated with each HDL subclass relative to the total HDL protein, determined during the pre- and post-supplementation periods with MiPo. Wilcoxon test, ^a^ *p* ≤ 0.05 vs. 0 h pre-treatment, ^b^ *p* ≤ 0.05 vs. 4 h pre-treatment, ^c^ *p* ≤ 0.05 vs. 8 h pre-treatment.

## Supplementary Materials

The following are available online at https://www.mdpi.com/2072-6643/11/8/1710/s1, Table S1: Anthropometric characteristics of the non-ACS women and nutrients intake before and after the supplementation with MiPo, Table S2: Effects of MiPo supplementation on plasma lipids and glucose concentrations in non-ACS women.
