# Supplementary material for: Microencapsulated Pomegranate Reverts High-Density Lipoprotein (HDL)-Induced Endothelial Dysfunction and Reduces Postprandial Triglyceridemia in Women with Acute Coronary Syndrome
Source: Nutrients. 2019 Jul 25;11(8):1710. doi: 10.3390/nu11081710 (PMC6722536; doi:10.3390/nu11081710)
Supplement: Supplementary file 1 [file nutrients-11-01710-s001.pdf]

Supplementary Table S1. Anthropometric characteristics of the non-ACS women and nutrients intake before and after 30 days of supplementation with MiPo

Parameters	Pre-supplementation Fasting condition n=6	Post-supplementation Fasting condition n=6	*P* value *
**Age (years)**	57 (41-65)		
**BMI (kg/m²)**	27.8 (25.4-30.1)	27.8 (25.1-29.8)	0.138
**Waist circumference (cm)**	93.7 (84.4-98.3)	92.9 (82.5-97.6)	0.027
**Systolic BP (mmHg)**	109 (100-117)	109 (99-113)	0.686
**Diastolic BP (mmHg)**	72 (63-75)	75 (70-82)	0.173
**Nutrients intake**			
**Carbohydrates (%)**	44.5 (41.2-47.9)	45.2 (43.0-46.9)	0.580
**Lipids (%)**	21.3 (18.9-22.4)	22.0 (19.7-23.6)	0.705
**Protein (%)**	30.2 (29.5-32.8)	29.4 (26.9-31.8)	0.105
**Calories (kcal)**	1765.7 (1641.9-1901.5)	1801.8 (1674.4-1899.7)	0.456

Data are expressed as median (interquartile range). * Wilcoxon's test. BMI: Body mass index. BP: Blood pressure

Supplementary Table S2. Effects of MiPo supplementation on plasma lipids and glucose concentrations in non-ACS women

Parameter		Sample time (n=6)		
		**0h**	**4h**	**8h**
**Glucose**	Pre	92.2 (86.6-100.8)	80.1 (71.1-93.8)	89.4 (83.7-91.3)
**(mg/dL)**	Post	83.8 (79.5-91.6)	93.6 (83.3-102.8)	89.8 (86.9-91.6)
**Total cholesterol**	Pre	208.0 (149.1-281.8)	190.5 (156.9-296.7)	232.1 (165.8-276.9)
**(mg/dL)**	Post	135.2 (114.6-172.0)[a]	153.8 (129.3-213.8)[d]	159.1 (110.3 -204.3)
**LDL-C**	Pre	130.4 (87.9-226.1)	112.2 (76.6-230.2)	135.2 (86.9-191.4)
**(mg/dL)**	Post	80.7 (63.4-107.9)[a]	82.1 (65.0-144.2)	91.5 (58.3-143.5)
**Triglycerides**	Pre	136.9 (89.0-235.5)	226.9 (130.4-306.1)[a]	267.4 (135.9-380.5)[a]
**(mg/dL9**	Post	95.6 (77.7-115.6)[a]	171.8 (146.9-189.3)[d]	145.7 (118.6-179.4)[d,e]
**iAUC**	Pre		471.4 (259.7-686.4)	
**(h.mg/dL)**	Post		404.7 (193.2-499.1)	
**HDL-C**	Pre	37.6 (34.4-40.5)	31.4 (29.0-33.4)[a]	34.0 (28.2-37.5)
**(mg/dL)**	Post	35.6 (32.1-41.3)	34.8 (31.9-38.3)	31.3 (27.6-37.8)[d]
**HDL-Tg**	Pre	27.3 (18.2-32.0)	30.9 (26.6-35.1)[a]	33.0 (30.9-36.1)[a]
**(mg/dL)**	Post	25.6 (23.1-28.6)	27.6 (22.1-34.0)	29.5 (20.9-37.0)
**HDL-Pho**	Pre	80.2 (66.3-102.4)	85.9 (79.4-97.5)	99.6 (89.8-111.5)[a,b]
**(mg/dL)**	Post	82.4 (65.8-99.4)	84.8 (71.7-93.2)	84.1 (75.1-96.9)[c]
**Ratios**				
**HDL-C/HDL-Pho**	Pre	0.469 (0.370-0.551)	0.349 (0.295-0.412)[a]	0.334 (0.297-0.357)[a]
	Post	0.442 (0.391-0.482)	0.429 (0.402-0.475)[b]	0.385 (0.275-0.399)[e]
**HDL-Tg/HDL-Pho**	Pre	0.358 (0.271-0.390)	0.356 (0.286-0.430)	0.333 (0.276-0.391)
	Post	0.296 (0.274-0.343)	0.364 (0.237-0.452)	0.369 (0.275-0.399)

The duration of the supplementation was 30 days. Data are expressed in median (interquartile range). Wilcoxon's test [a] p≤0.05 *vs.* 0h pre-supplementation. [b] p≤0.05 *vs.* 4h pre-supplementation. [c] p≤0.05 *vs.* 8h pre-supplementation. [d] p≤0.05 *vs.* 0h post-supplementation. [e] p≤0.05 *vs.* 4h post-supplementation.
